# The causal effect of obesity on diabetic retinopathy: A two-sample Mendelian randomization study

**DOI:** 10.3389/fendo.2023.1108731

**Published:** 2023-04-03

**Authors:** Changwei Zheng, Xin Wei, Xiaochuan Cao

**Affiliations:** Department of Ophthalmology, The People’s Hospital of Tongliang District, Chongqing, China

**Keywords:** obesity, diabetic retinopathy, body mass index, Mendelian randomization, waist circumference

## Abstract

**Background:**

The causal effect of obesity on diabetic retinopathy (DR) remains controversial. The aim of this study was to assess the causal association of generalized obesity evaluated by body mass index (BMI) and abdominal obesity evaluated by waist or hip circumference with DR, background DR, and proliferative DR using a two-sample Mendelian randomization (MR) analysis.

**Methods:**

Genetic variants associated with obesity at the genome-wide significance (P<5×10^−8^) level were derived using GWAS summary statistics from the UK Biobank (UKB) with a sample size of 461 460 individuals for BMI, 462 166 individuals for waist circumference, and 462 117 individuals for hip circumference. We obtained genetic predictors of DR (14 584 cases and 202 082 controls), background DR (2026 cases and 204 208 controls), and proliferative DR (8681 cases and 204 208 controls) from FinnGen. Univariable and multivariable Mendelian randomization analyses were conducted. Inverse variance weighted (IVW) was the main method used to analyze causality, accompanied by several sensitivity MR analyses.

**Results:**

Genetically predicted increased BMI [OR=1.239; 95% CI=(1.134, 1.353);P=1.94×10^-06^], waist circumference [OR=1.402; 95% CI=(1.242, 1.584); P=5.12×10^-08^], and hip circumference [OR=1.107; 95% CI=(1.003, 1.221); P=0.042] were associated with increased risk of DR. BMI [OR=1.625; 95% CI=(1.285, 2.057); P=5.24×10^-05^], waist circumference [OR=2.085; 95% CI=(1.54, 2.823); P=2.01×10^-06^], and hip circumference [OR=1.394; 95% CI=(1.085, 1.791); P=0.009] were correlated with the risk of background DR. MR analysis also supported a causal association between BMI [OR=1.401; 95% CI=(1.247, 1.575); P=1.46×10^-08^], waist circumference [OR=1.696; 95% CI=(1.455, 1.977); P=1.47×10^-11^], and hip circumference [OR=1.221; 95% CI=(1.076, 1.385); P=0.002] and proliferative DR. The association of obesity with DR continued to be significant after adjustment for type 2 diabetes.

**Conclusion:**

This study using two-sample MR analysis indicated that generalized obesity and abdominal obesity might increase the risk of any DR. These results suggested that controlling obesity may be effective in DR development.

## 1 Introduction

Diabetic retinopathy (DR), which is a microvascular diabetic complication, remains one of the leading preventable causes of visual impairment and blindness worldwide. Almost all type 1 diabetes patients and 60% of type 2 diabetes patients develop retinopathy within 20 years (1). It is estimated that the number of DR cases will reach 191 million, and without timely intervention and treatment, 56.6 million patients will develop vision-threatening DR by 2030 (2). Moreover, even with strict glucose regulation, some patients with type 2 diabetes still develop DR after 6.5–13.3 years (3). Therefore, studies to identify other modifiable risk factors for DR are essential to guide clinical practice to prevent DR occurrence and progression (4).

Obesity (defined as a body mass index (BMI) ≥30 kg/m^2^) is an emerging public health problem and a widely accepted risk factor for many diseases, such as type 2 diabetes, cardiovascular diseases (CVD), and cancer (5–7). Various studies have reported the effects of obesity on the risk of DR (8), but the causal association between obesity and DR remains controversial. According to the World Health Organization (WHO) classification, there are two types of obesity: general obesity assessed by BMI and abdominal obesity assessed by waist circumference, hip circumference, or waist-to-hip ratio (WHR) (7). Western studies have reported a significant association between higher BMI and any stage of DR (9, 10). In contrast, some studies conducted in Asian populations demonstrated no significant BMI-DR associations (11) and even inverse BMI-DR associations (12, 13). Therefore, there still seems to be an “obesity paradox” between obesity and DR (14). The term “obesity paradox” was originally used to describe the finding that being overweight or even obese is “protective” of or has no impact on CVD and mortality (15). Similarly, equivocal results have been obtained for the association between abdominal obesity and DR. WHR was reported to be positively associated with any stage of DR (13, 16) or to have no significant association (17). In a recent longitudinal cohort study, WHR was also connected with an increased risk of incident DR in a 2-year follow-up (18). Therefore, whether obesity causes protective or detrimental effects on DR needs to be further clarified. Furthermore, it is of critical importance to determine whether obesity is an independent risk factor for DR, as it is potentially modifiable.

Compared to traditional retrospective studies, Mendelian randomization (MR) studies are less affected by confounding factors, and the causal sequence is more reasonable (19). This approach treats genetic variations as a “natural” randomized controlled trial in which individuals are randomly assigned to different exposure levels over their lifetime, which has achieved great success in finding risk factors for many diseases (20). However, to our knowledge, no MR study has been used to evaluate the effects of obesity on the risk of DR. The present study used a two-sample MR approach to explore the causal relationship between obesity and DR, which may provide guidance on the prevention and treatment of DR.

## 2 Methods

### 2.1 Study design and instrumental variable extraction

We reported the MR study in adherence to the Strengthening the Reporting of Observational Studies in Epidemiology using Mendelian Randomization (STROBE-MR) (21). The MR study was analyzed using recent genome-wide association study (GWAS) summary statistics, and ethical approval was obtained from the respective institutions. A two-sample MR analysis was used to explore the causal relationships between obesity and DR. Type 2 diabetes is the most important risk factor for DR. Meanwhile, the genetic overlap between obesity and diabetes is widespread (22), and we used multivariable MR (MVMR) to mitigate potential pleiotropic effects *via* diabetes.

**Figure 1:**
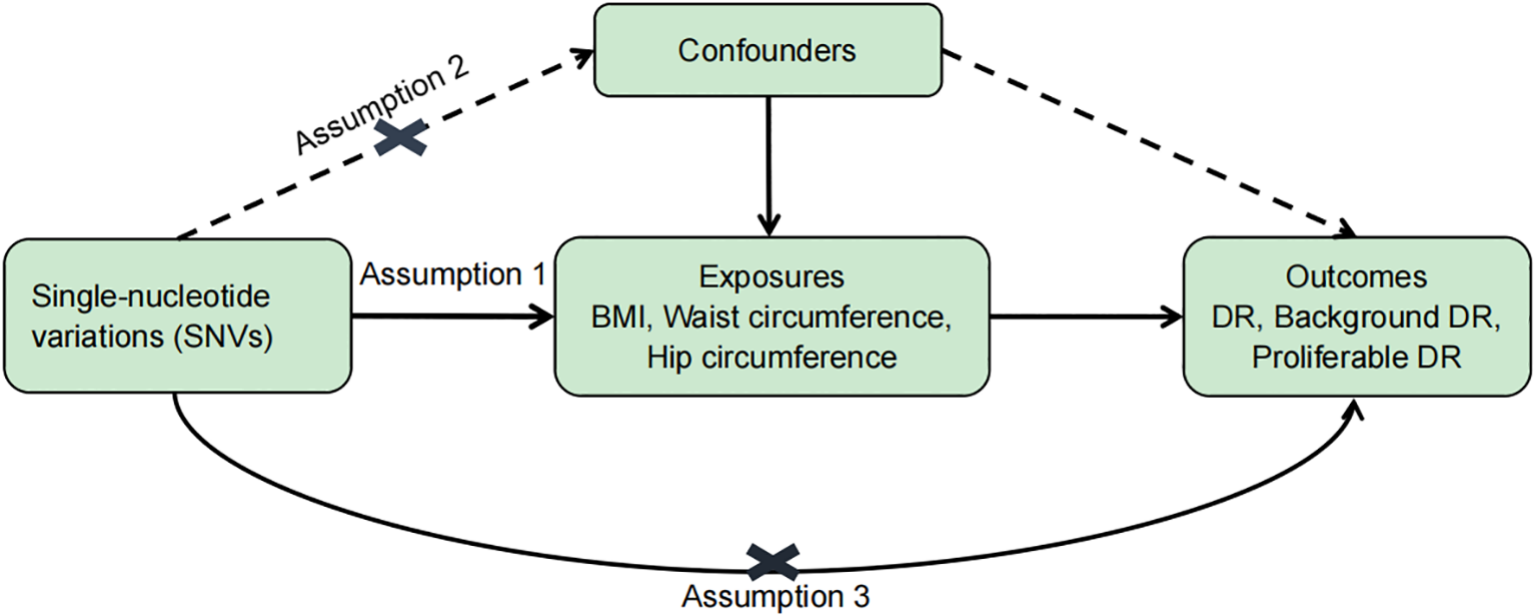
Basic assumptions of Mendelian randomization. Assumption 1: SNVs were closely associated with exposure. Assumption 2: SNVs were not associated with any potential confounders. Assumption 3: SNVs are only linked to the outcome through exposure.

To evaluate the causal relationship between obesity (BMI, waist circumference, and hip circumference) and DR, single-nucleotide variations (SNVs) were selected according to the following criteria (**Figure 1**): (1) SNVs were closely associated with exposure and reached genome-wide significance (*p* < 5 × 10^−8^); (2) SNVs were not associated with any potential confounders and were independent of each other to avoid biases caused by linkage disequilibrium (*r* ^2^ < 0.0001, clumping distance = 10,000 kb); and (3) SNVs are only linked to the outcome through exposure. An *F* statistic (*F* = beta^2^/se^2^; beta for the SNV-exposure association (beta); variance (se)) was calculated for each SNV (23). Since an empirical threshold of more than 10 indicates that the SNV has sufficient validity, SNVs with an *F* statistic of less than 10 were removed. MR–Steiger filtering was used to remove variations that were more strongly correlated with DR than with obesity (24). Information on the *F* statistic, SNVs, and MR–Steiger is provided in **Supplementary Datasets 1**–**9**.

### 2.2 Data sources

In the present work, we chose obesity-associated indices (BMI, waist, and hip circumference) from the UK Biobank (UKB) as exposures. UKB was a UK-based cohort study that recruited about 500,000 participants aged 40–69 years between 2006 and 2010, from whom a series of medical and physical information was collected (25). BMI is the ratio of weight in kilograms divided by the squared height in meters. The natural indent was measured for the waist circumference. The widest part of the hip was recorded for the hip circumference. To reduce confounding by race, we only used summary statistics from individuals of European descent with a sample size of 461,460 individuals for BMI, 462,166 individuals for waist circumference, and 462,117 individuals for hip circumference, and it is available for download (https://gwas.mrcieu.ac.uk/). Different stages of DR (DR, background DR, and proliferative DR) were chosen as outcomes. The GWAS summary statistics of DR were extracted from the FinnGen (https://r5.finngen.fi/). Participants in the DR (GWAS ID: finn-b-DM_RETINOPATHY) analysis included 14,584 cases and 202,082 controls; participants in the background DR (GWAS ID: finn-b-DM_BCKGRND_RETINA) analysis included 2,026 cases and 204,208 controls; and participants in the proliferative DR (GWAS ID: finn-b-DM_RETINA_PROLIF) analysis included 8,681 cases and 204,208 controls. Cases of different stages of DR were identified based on the International Classification of Diseases-Revision 9/10 criteria from the hospital discharge registry (https://r5.risteys.finngen.fi/). We obtained genetic predictors of type 2 diabetes from Mahajan et al. (26).

### 2.3 Statistical analyses

All statistical and MR analyses were performed using R software (version 4.1.1) using the R packages “TwoSampleMR” and “MR-PRESSO”. *p* < 0.05 was considered to be statistically significant as evidence for a potential causal association.

The inverse variance-weighted (IVW) method was used as the primary method for calculating the causal effect. Given that the validity of the MR method is strictly dependent on the absence of pleiotropy, we used a series of MR analytical approaches to account for pleiotropy. First, we used MR–Egger (27) and weighted-median (WM) (28) methods as supplements. The better method between IVW and MR–Egger was selected *via* Ruecker’s framework. *p* < 0.05 of Cochran’s *Q* and Rucker’s *Q* (*Q*–*Q*) indicates the MR−Egger analysis with the least heterogeneity (29), which is reported in **Supplementary Table S1**. Second, we determined the heterogeneity of different genetic variants using Cochrane’s *Q* test and *I* ^2^. *p* < 0.05 of Cochrane’s *Q (* 30) and *I* ^2^ > 25% (31) were considered to indicate significant heterogeneity. Next, the pleiotropic effect of the genetic variants was assessed using the MR–Egger intercepts (32) and MR-PRESSO global test (33). In addition to these methods, MR–Steiger filtering was used to remove variations that were more strongly correlated with DR than with obesity. Finally, the SNV leave-one-out method was used to further verify the robustness of the data (**Supplementary Figures S1**–**S9**).

## 3 Results

**Figure 2:**
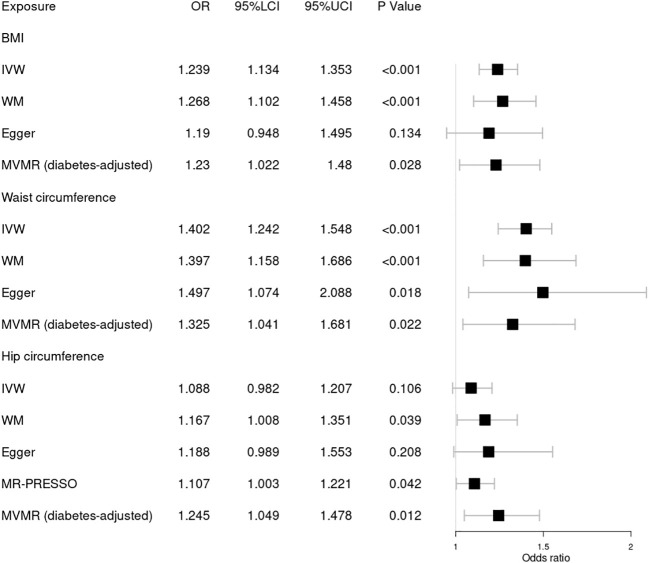
Forest plot of Mendelian randomization results of obesity effect on DR. BMI, body mass index; IVW, inverse variance weighted; Egger, MR–Egger; WM, weighted-median; MR-PRESSO, the outlier-corrected MR pleiotropy residual sum and outlier results (one significant outlier; SNV:rs7903146); MVMR, multivariable Mendelian randomization; 95% LCI, lower limit of 95% CI; 95% UCI, upper limit of 95% CI.

Our results indicated that 305, 252, and 275 SNVs in DR were associated with BMI and waist and hip circumference, respectively. A total of 306, 252, and 275 SNVs in background DR were associated with BMI, waist circumference, and hip circumference, while 305, 252, and 274 SNVs in proliferative DR were associated with BMI, waist circumference, and hip circumference, respectively (**Table 1**). The *F* statistic of each SNV was greater than the empirical threshold of 10, and the minimum *F* statistics in subgroups are shown in **Table 1**. The explained variances ranged from 2.51% to 4.21% for different stages of DR. The main results of the MR analysis are presented in **Figures 2** **–** **4**, and more details are provided in **Supplementary Table S2**.

**Table 1:** Mendelian randomization results of obesity traits on DR.

Exposures	Outcomes	NSNVs	*F* statistic	*R* ^2^ (%)	*I* ^2^ (%)	Cochrane’s *Q*		MR–Egger test		MR-PRESSO
						*Q*	*p*-value	Intercept	*p*-value	*p*-value
BMI	DR	305	29.88	4.2	0	301.82	5.25E−01	8.16E−04	0.708	5.35E−01
Waist circumference		252	29.76	2.51	14.23	292.95	3.60E−02	−1.12E−03	0.679	4.20E−02
Hip circumference		276	29.85	3.99	24.4	363.76	2.62E−04	−1.84E−03	0.489	1.67E−04
Hip circumference^a^		275	29.85	3.98	16.52	328.21	1.40E−02	−1.87E−03	0.458	7.98E−01
BMI	Background DR	306	29.88	4.21	8.84	334.57	1.18E−01	4.71E−03	0.429	1.25E−01
Waist circumference		252	29.76	2.51	10.01	278.93	1.09E−01	−4.66E−03	0.489	1.09E−01
Hip circumference		276	29.85	3.99	21.01	348.17	2.00E−03	−2.54E−03	0.702	2.00E−03
Hip circumference^b^		275	29.85	3.98	16.69	328.89	1.30E−02	−2.45E−03	0.704	8.40E−01
BMI	Proliferative DR	305	29.88	4.2	6.99	326.84	1.76E−01	2.35E−03	0.417	1.71E−01
Waist circumference		252	29.76	2.51	12.06	285.42	6.70E−02	−3.82E−04	0.911	6.90E−02
Hip circumference		276	29.85	3.99	27.5	379.33	3.00E−05	−1.87E−03	0.589	1.67E−04
Hip circumference^c^		274	29.85	3.96	17.85	332.31	8.00E−03	−1.88E−03	0.564	6.92E−01

DR, diabetic retinopathy; NSNVs, number of single-nucleotide variations; BMI, body mass index; R^2^, phenotype variance explained by genetics. ^a^One significant outlier (SNV:rs7903146) was deleted. ^b^One significant outlier (SNV:rs35506085) was deleted. ^c^Two significant outliers (SNV:rs35506085; SNV:rs7903146) were deleted.

**Figure 3:**
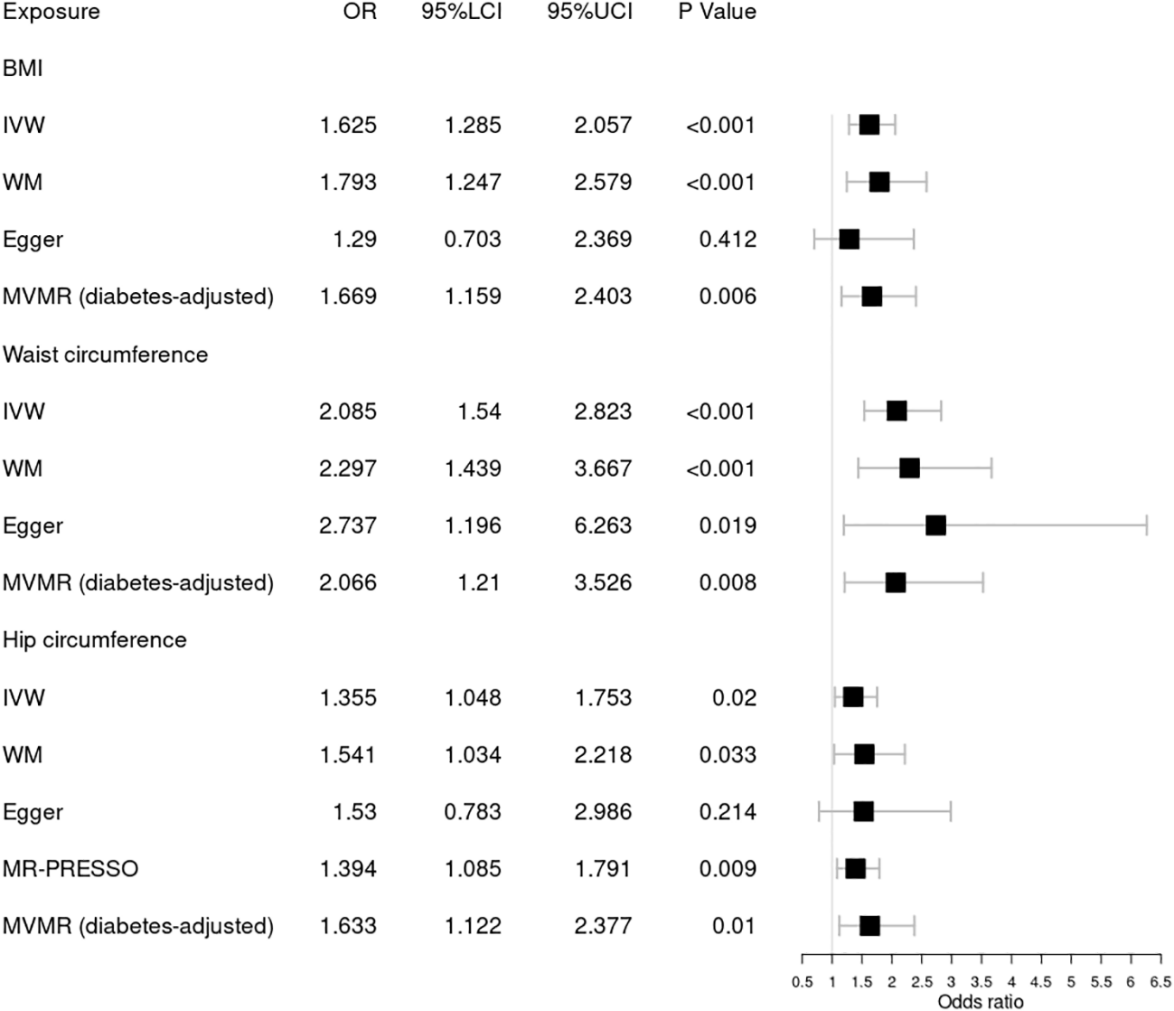
Forest plot of Mendelian randomization results of obesity effect on background DR. BMI, body mass index; IVW, inverse variance weighted; Egger, MR–Egger; WM, weighted-median; MR-PRESSO, the outlier-corrected MR pleiotropy residual sum and outlier results (one significant outlier, SNV:rs35506085); MVMR, multivariable Mendelian randomization; 95% LCI, lower limit of 95% CI; 95% UCI, upper limit of 95% CI.

**Figure 4:**
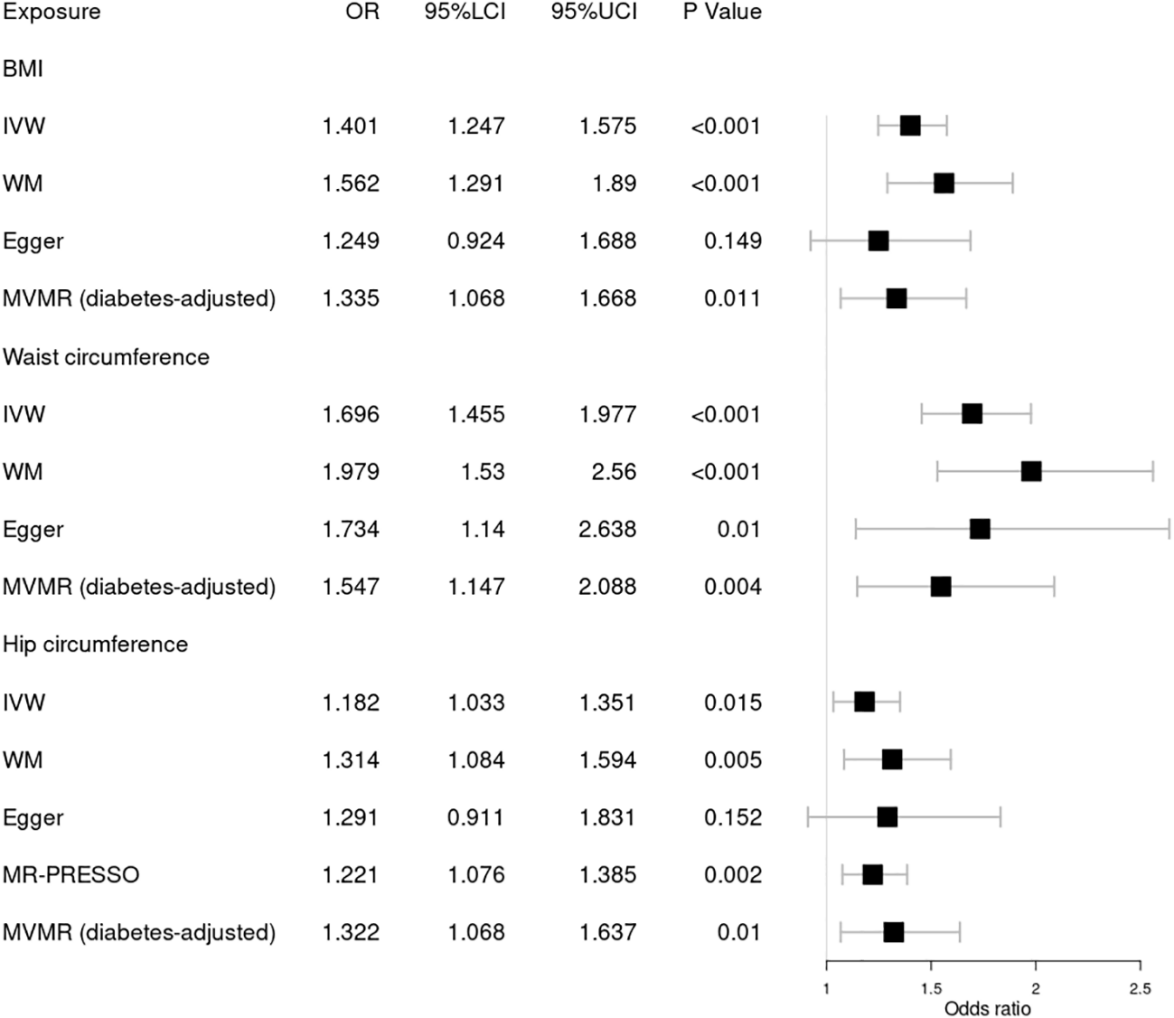
Forest plot of Mendelian randomization results of obesity effect on proliferative DR. BMI, body mass index; IVW, inverse variance weighted; Egger, MR–Egger; WM, weighted-median; MR-PRESSO, the outlier-corrected MR pleiotropy residual sum and outlier results (two significant outliers, SNV:rs35506085; SNV:rs7903146); MVMR, multivariable Mendelian randomization; 95% LCI, lower limit of 95% CI; 95% UCI, upper limit of 95% CI.

### 3.1 Causal effect of obesity on DR

First, we explored the causal relationship between obesity and DR, as shown in **Figure 2**. Genetically predicted higher BMI [OR = 1.239; 95% CI = (1.134, 1.353); *p* = 1.94 × 10^−06^] and waist circumference [OR = 1.402; 95% CI = (1.242, 1.584); *p* = 5.12 × 10^−08^] by the IVW method were significantly associated with a higher risk of DR, consistent with results obtained by WM. Nonsignificant pleiotropy in BMI was detected by Cochrane’s *Q* test (*p* = 0.525), *I* ^2^ = 0, MR–Egger intercept (*p* = 0.708), or MR-PRESSO global test (*p* = 0.535). Slight heterogeneity was present in waist circumference (*Q* = 292.95; *p* = 0.036), but no significant outlier (*p* < 0.05) was identified by MR-PRESSO. Significant heterogeneity in hip circumference was detected by Cochrane’s *Q* test (*p* = 2.62 × 10^−04^), and a significant outlier (SNV:rs7903146) was detected by MR-PRESSO. Higher hip circumference was also suggestively associated with the risk of DR using the IVW method [OR = 1.107; 95% CI = (1.003, 1.221); *p* = 0.042] after deleting the outlier. Moreover, our MVMR analysis suggested that the causal association between obesity and DR existed apart from diabetes. Using the MR–Steiger test, none of the variants were removed, and the results remained unchanged. Finally, the leave-one-out analysis found that no single SNV strongly drove the overall effect of obesity on DR.

### 3.2 Causal effect of obesity on background DR

Next, we assessed the causal relationship between obesity and background DR, as shown in **Figure 3**. IVW analysis indicated that genetically predicted increased BMI [OR = 1.625; 95% CI = (1.285, 2.057); *p* = 5.24 × 10^−05^], waist circumference [OR = 2.085; 95% CI = (1.54, 2.823); *p* = 2.00 × 10^−06^], and hip circumference [OR = 1.394; 95% CI = (1.085, 1.791); *p* = 0.009] were associated with a higher risk of background DR. The WM method showed similar results. Pleiotropy in BMI and waist circumference identified by Cochrane’s *Q* test, *I* ^2^, MR–Egger intercept, and MR-PRESSO did not reach statistical significance (all *p* > 0.05 or *I* ^2^ < 25%). However, there was significant pleiotropy (MR-PRESSO, *p* = 0.002) in hip circumference, and MR-PRESSO detected one significant outlier (SNV:rs35506085). The IVW and WM methods still suggested a causal association between the hip circumference and background DR after deleting the outlier. The associations for obesity and background DR remained significant after adjustment for type 2 diabetes through MVMR. No one SNV was excluded by MR–Steiger. Additionally, the leave-one-out test showed that the MR results were not significantly affected by a single SNV.

### 3.3 Causal effect of obesity on proliferative DR

Finally, we further investigated the relationship between obesity and the risk of proliferative DR using MR analysis, as shown in **Figure 4**. Higher BMI [OR = 1.401; 95% CI = (1.247, 1.575); *p* = 1.46 × 10^−08^], waist circumference [OR = 1.696; 95% CI = (1.455, 1.977); *p* = 1.47 × 10^−11^], and hip circumference [OR = 1.221; 95% CI = (1.076, 1.385); *p* = 0.002] were suggestively associated with the increasing risk of proliferative DR using the IVW method. These results were supported by those of the WM method. No significant pleiotropy in BMI or waist circumference was detected by several sensitivity MR analyses. However, significant pleiotropy in hip circumference was found by Cochrane’s *Q* test (*p* = 3.00 × 10^−05^) or *I* ^2^ = 27.5%. MR-PRESSO found two significant outliers (SNV:rs35506085; SNV:rs7903146). After deleting the two outliers, the causal relationship still persisted. Meanwhile, MVMR analysis indicated a causal relationship between obesity and proliferative DR aside from diabetes. Finally, MR–Steiger and the SNV leave-one-out method were used to further validate the data robustness, and no SNV was excluded.

## 4 Discussion

The present study investigated the causal association between generalized obesity (evaluated by BMI) and abdominal obesity (evaluated by waist and hip circumference) with different stages of DR using MR analysis. This study corroborated the conclusion that both generalized obesity and abdominal obesity are risk factors for DR (including background and proliferative DR). After adjusting for diabetes by MVMR, the causal relationship between obesity and DR still exists, suggesting that obesity may be an independent risk factor for DR.

Obesity and diabetes are recognized as major public health problems worldwide. A series of scientific studies have indicated that obesity is involved not only in the pathogenesis of diabetes but also in the development of its complications (34). However, inconsistent conclusions on the association between generalized obesity or abdominal obesity and DR were reported in previous studies. In particular, some studies have shown an inverse BMI-DR association. In fact, BMI may be more susceptible to the impact of disease (35) than other obesity-associated indices. The “obesity paradox” is widely discussed in the association between obesity and CVD. Stamatina et al. strongly reaffirmed that being overweight heightens the risk of CVD and pointed out that the “obesity paradox” is mainly due to the effect of confounding on BMI (5). Therefore, the inverse BMI-DR association may be due to confounding BMI in these traditional studies.

A recent meta-analysis pooled only prospective cohort studies, providing a high level of evidence that a higher BMI significantly increases the risk of DR incidence (36). Our MR results supplied genetic evidence to support the notion that a higher BMI is a potential risk factor for any DR in individuals of European descent. To date, a few mechanisms may account for the deleterious effect of BMI on DR. First, an increase in BMI increases linearly with the risk of type 2 diabetes (37), which plays a key role in the pathogenesis of DR. Moreover, an elevated BMI is often correlated with hypertension and dyslipidemia, both of which are risk factors for DR (38). Second, high BMI exaggerates hyperglycemia-induced epigenetic modifications, leading to mitochondrial damage (39) and the development of DR (40). Additionally, ethnic differences should not be ignored when interpreting the relationship between BMI and DR (41).

BMI has limited value in accounting for fat distribution, as abdominal fat (i.e., waist circumference) is more strongly correlated with visceral fat than BMI (42). Abdominal obesity may be a more critical factor of DR and was positively associated with all stages of DR (13), which was supported by our MR results. Increasing waist circumference was causally associated with a higher risk of any DR. The consistent results of the MR analyses (IVW, WM, and MR–Egger) indicated that the conclusion was robust and reliable. A recent longitudinal cohort study also supported this relationship and indicated that abdominal obesity increased the risk of 2-year incident DR (18). The pathophysiological mechanisms underlying the detrimental effect of abdominal obesity on DR are unclear. In fact, abdominal obesity may be mediated through the impact of visceral fat on adverse metabolic profiles, including insulin resistance and inflammation (43), which have been implicated in the pathogenesis of DR (44). Moreover, excess abdominal fat may disrupt the secretion of growth hormone (43), implicating pathological neovascularization in DR.

To the best of our knowledge, this is the first study that has applied MR analysis to investigate the potential causal association between obesity and the risk of DR. The first advantage of the study is the MR design, which mitigates bias from reverse causation and confounding. The second advantage is that our MR study strictly utilized European subjects, thus minimizing bias due to population stratification. This study also has several limitations. The greatest concern in MR studies is horizontal pleiotropy, which occurs when genetic variants influence the outcome of more than one pathway. We designed a series of MR analytical approaches to minimize this bias. However, it is not possible to completely rule out residual pleiotropy. Moreover, MR analysis only made the assumption of a linear relationship (20) between obesity and DR; thus, additional studies are needed to determine the underlying mechanism.

## 5 Conclusion

In conclusion, these findings demonstrated a causal relationship between obesity and DR. Our MR analysis showed that obesity may be an independent risk factor for different stages of DR, which suggested that controlling obesity may be effective in DR development.

## Data availability statement

The datasets presented in this study can be found in online repositories. The names of the repository/repositories and accession number(s) can be found in the article/**Supplementary Material**.

## Author contributions

CZ and XW performed the main data analysis and wrote the draft of the manuscript. XC supervised the whole research and is responsible for the integrity of the data analysis. All authors contributed to the article and approved the submitted version.
