# Supplementary material for: The causal effect of obesity on diabetic retinopathy: A two-sample Mendelian randomization study
Source: Front Endocrinol (Lausanne). 2023 Apr 3;14:1108731. doi: 10.3389/fendo.2023.1108731 (PMC10106681; doi:10.3389/fendo.2023.1108731)

Figure 1: Leave-one-out analysis plots for BMI on the risk of DR.

Figure 2: Leave-one-out analysis plots for waist circumference on the risk of DR.

Figure 3: Leave-one-out analysis plots for hip circumference on the risk of DR.

Figure 4: Leave-one-out analysis plots for BMI on the risk of background DR.

Figure 5: Leave-one-out analysis plots for wasit circumference on the risk of background DR.

Figure 6: Leave-one-out analysis plots for hip circumference on the risk of background DR.

Figure 7: Leave-one-out analysis plots for BMI on the risk of proliferative DR.

Figure 8: Leave-one-out analysis plots for wasit circumference on the risk of proliferative DR.

Figure 9: Leave-one-out analysis plots for hip circumference on the risk of proliferative DR.

1.
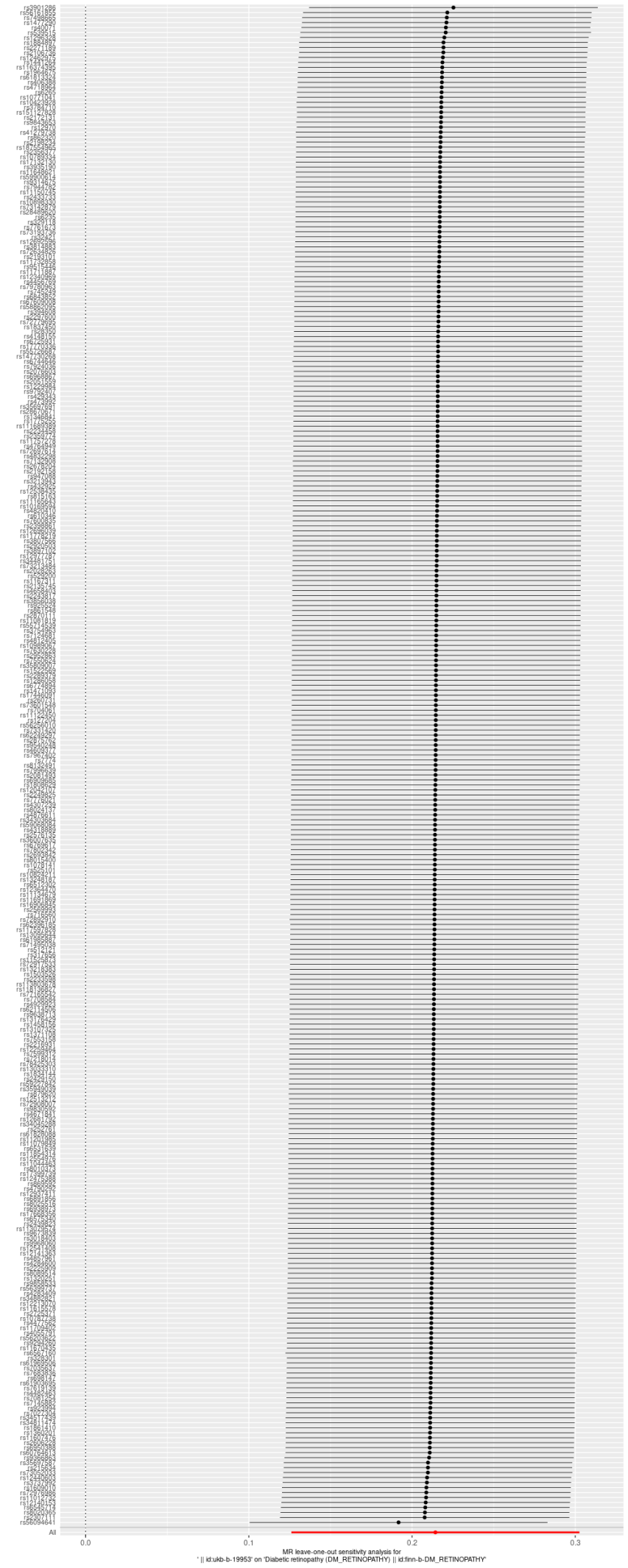

2.
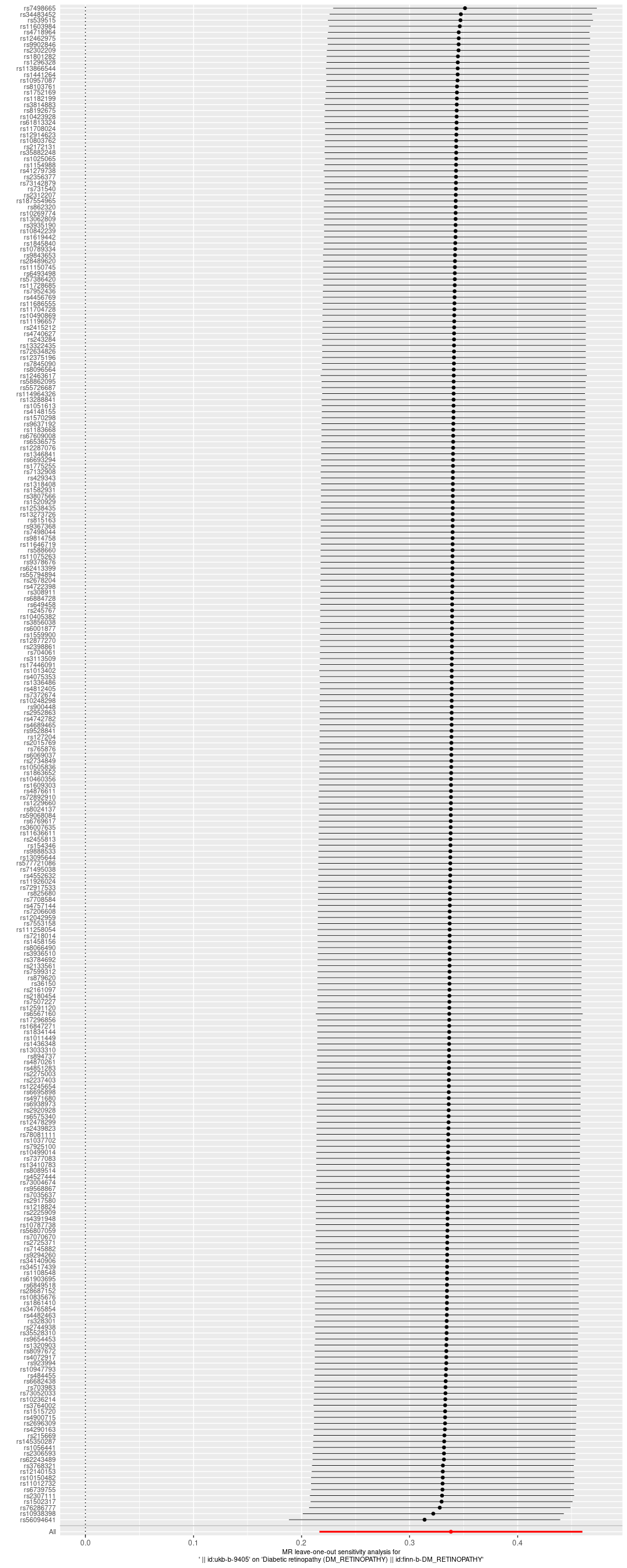

3.
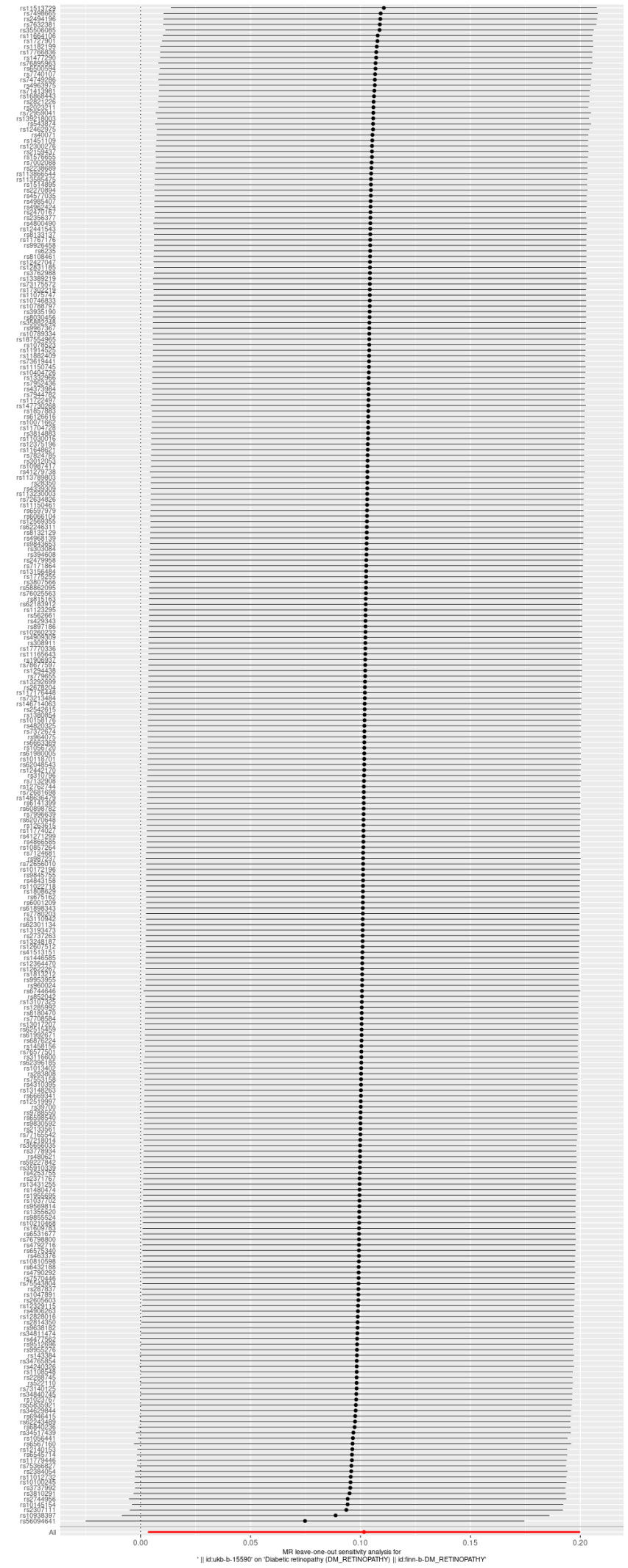

4.
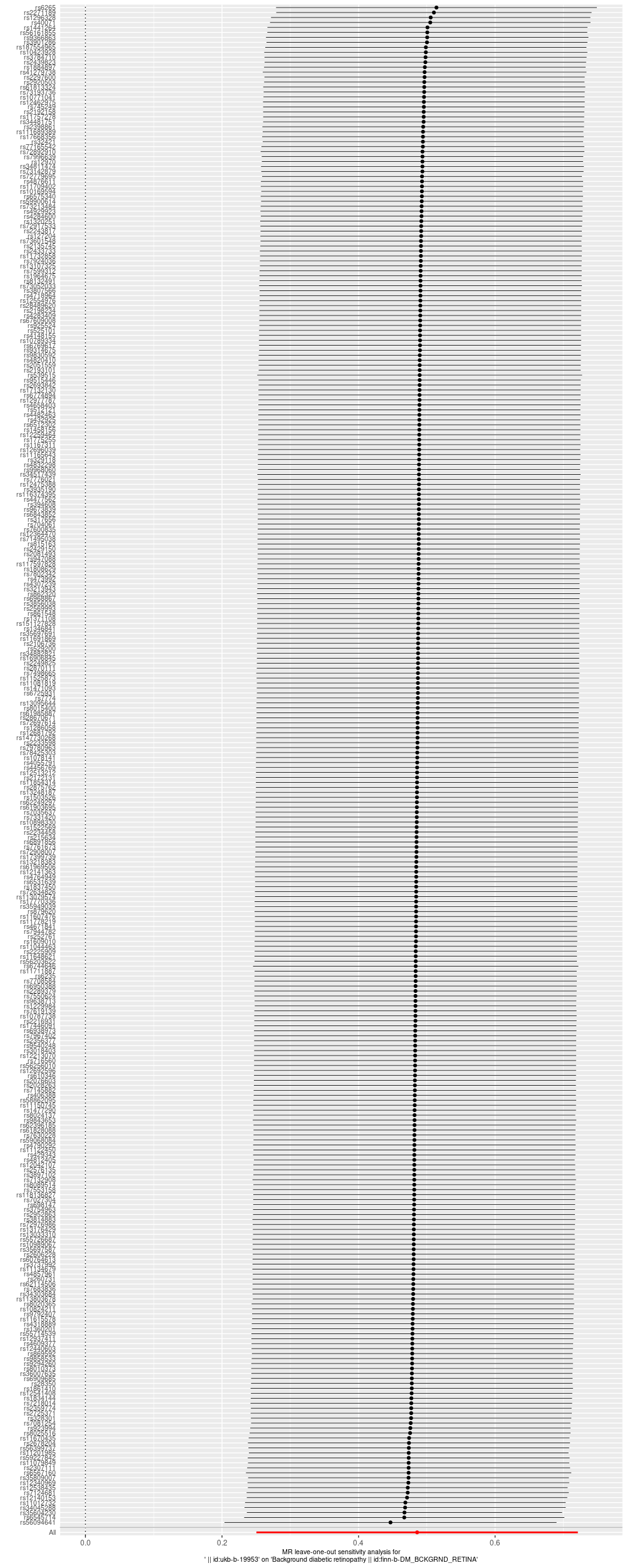

5.
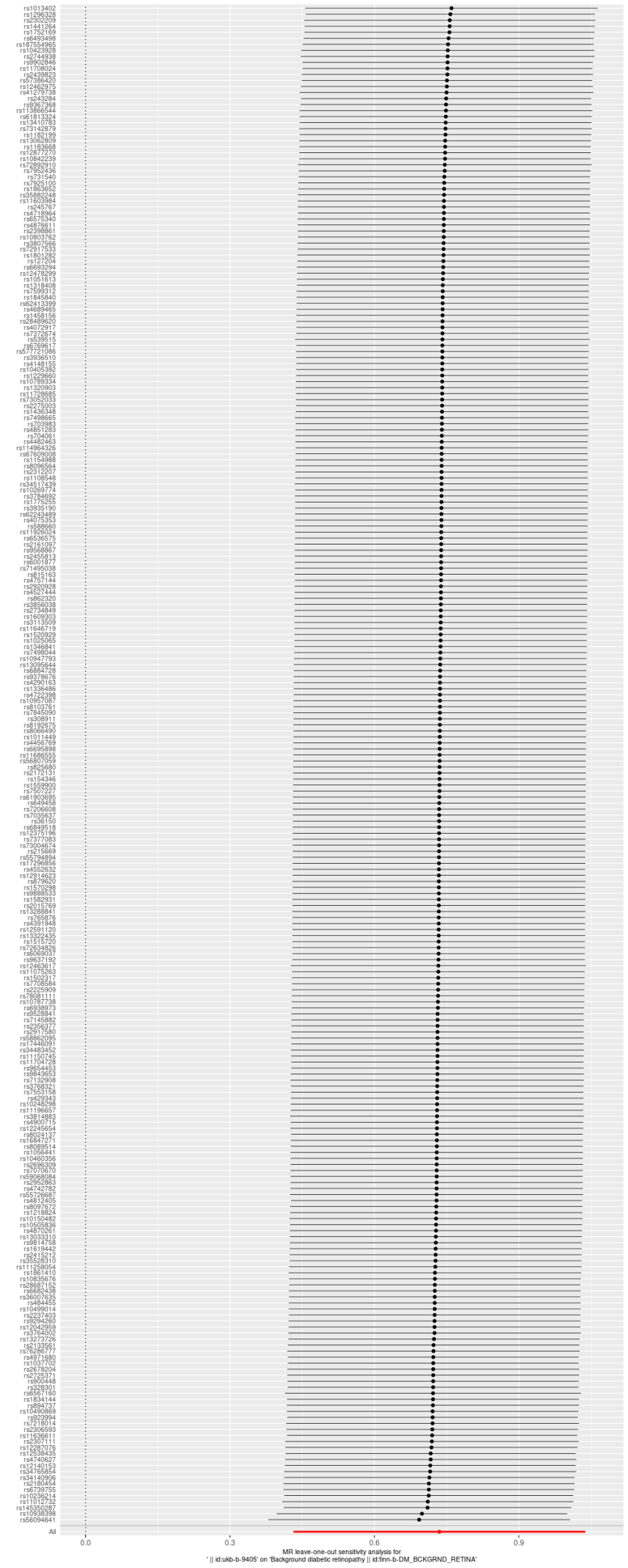

6.
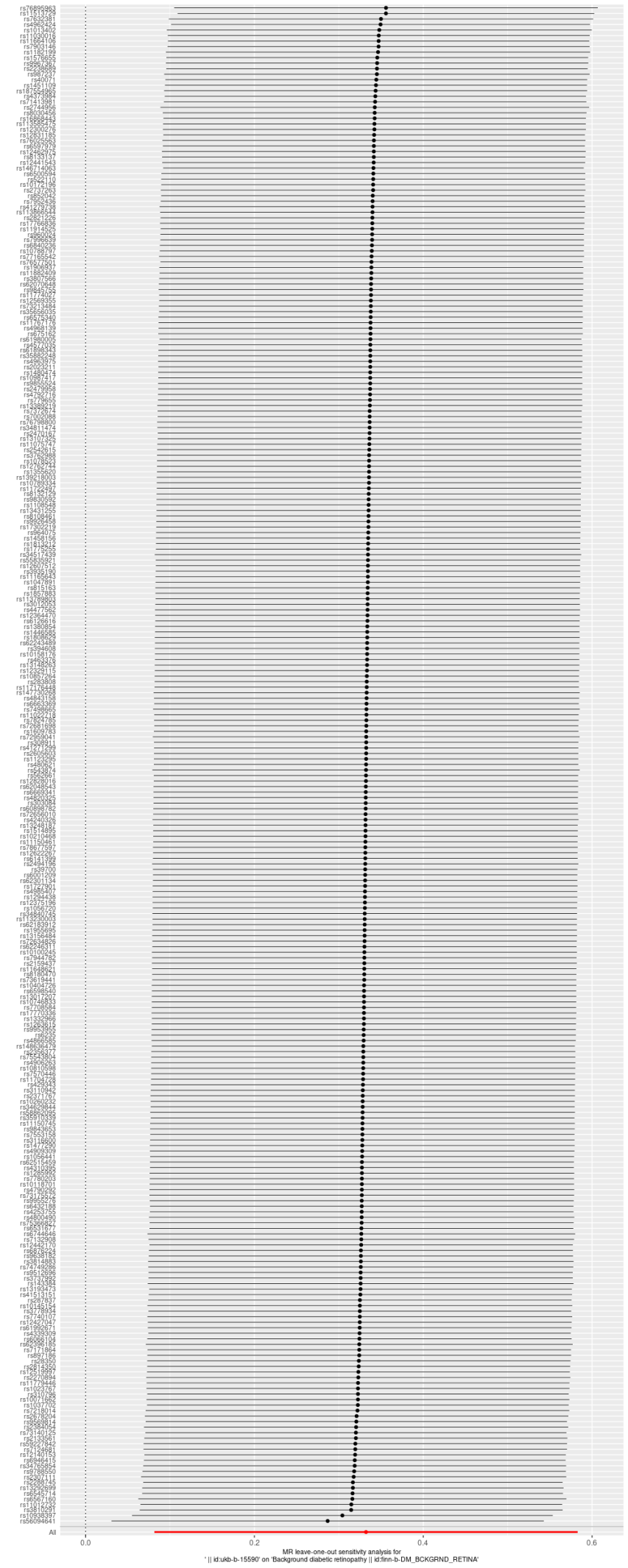

7.
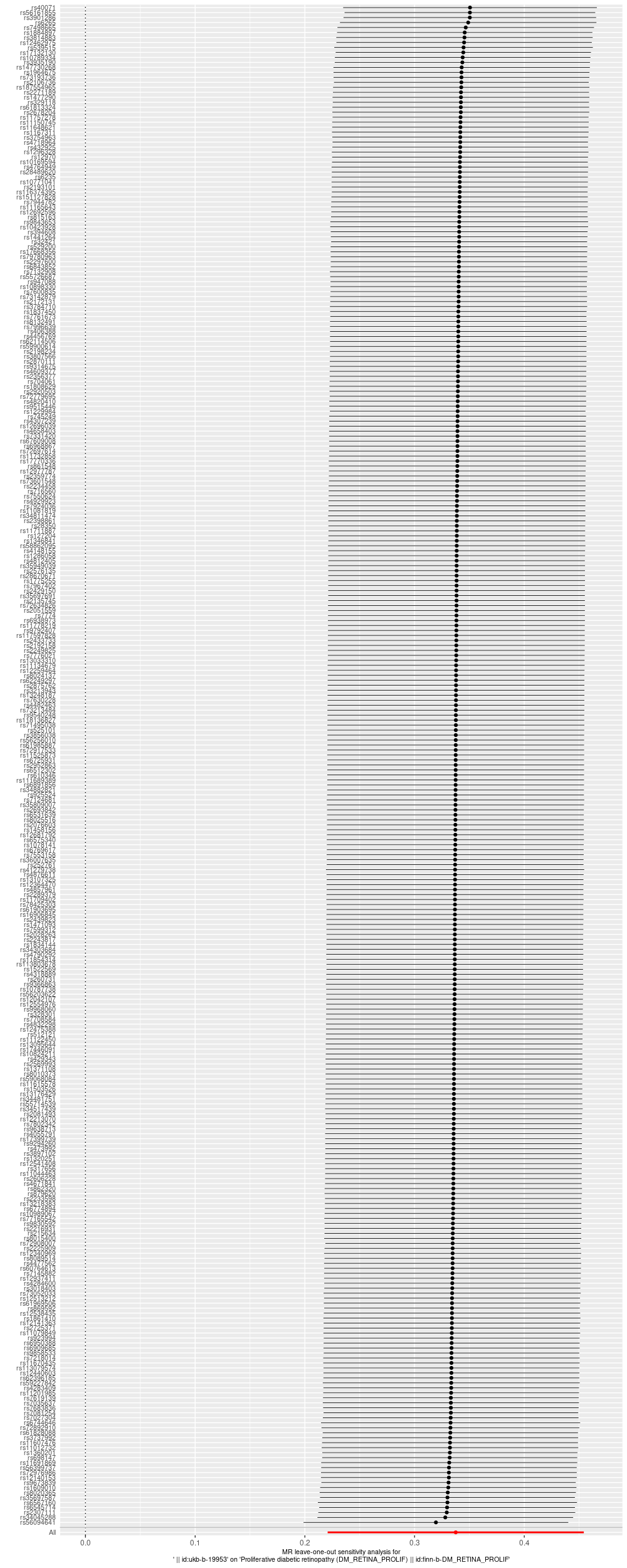

8.
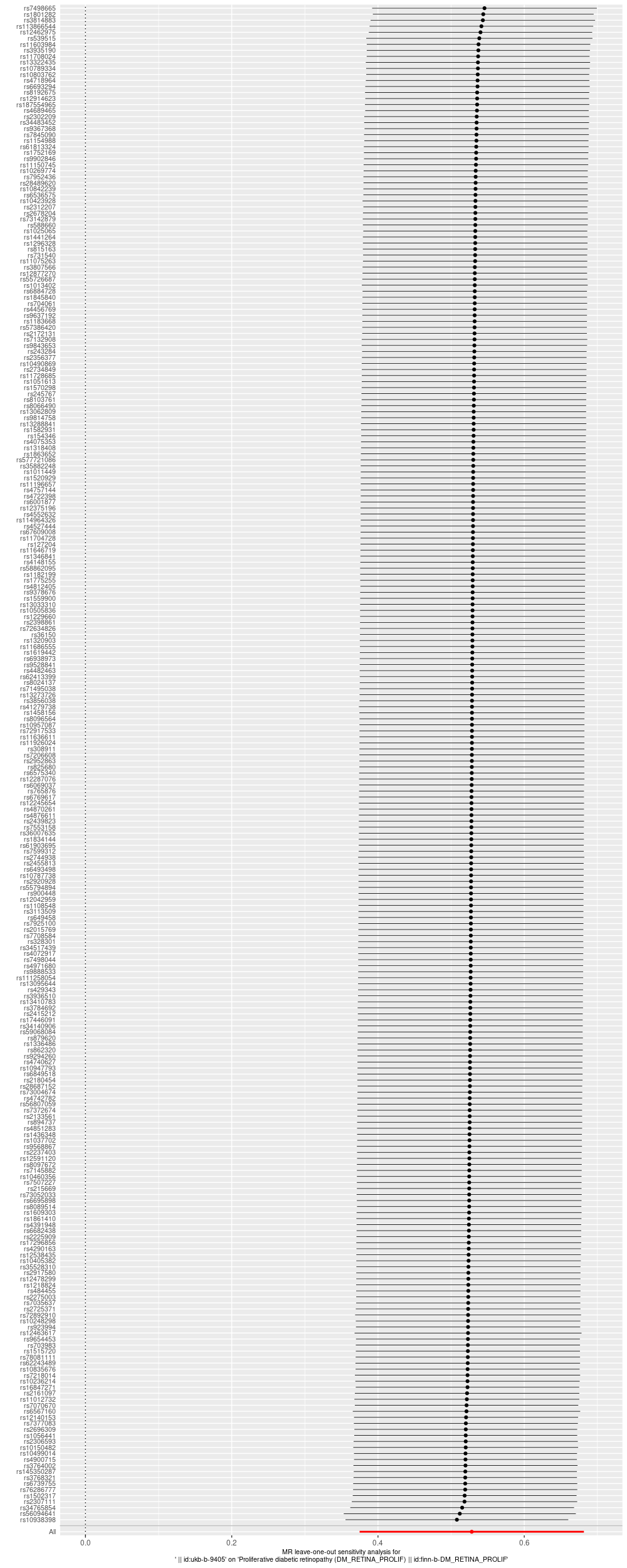

9.
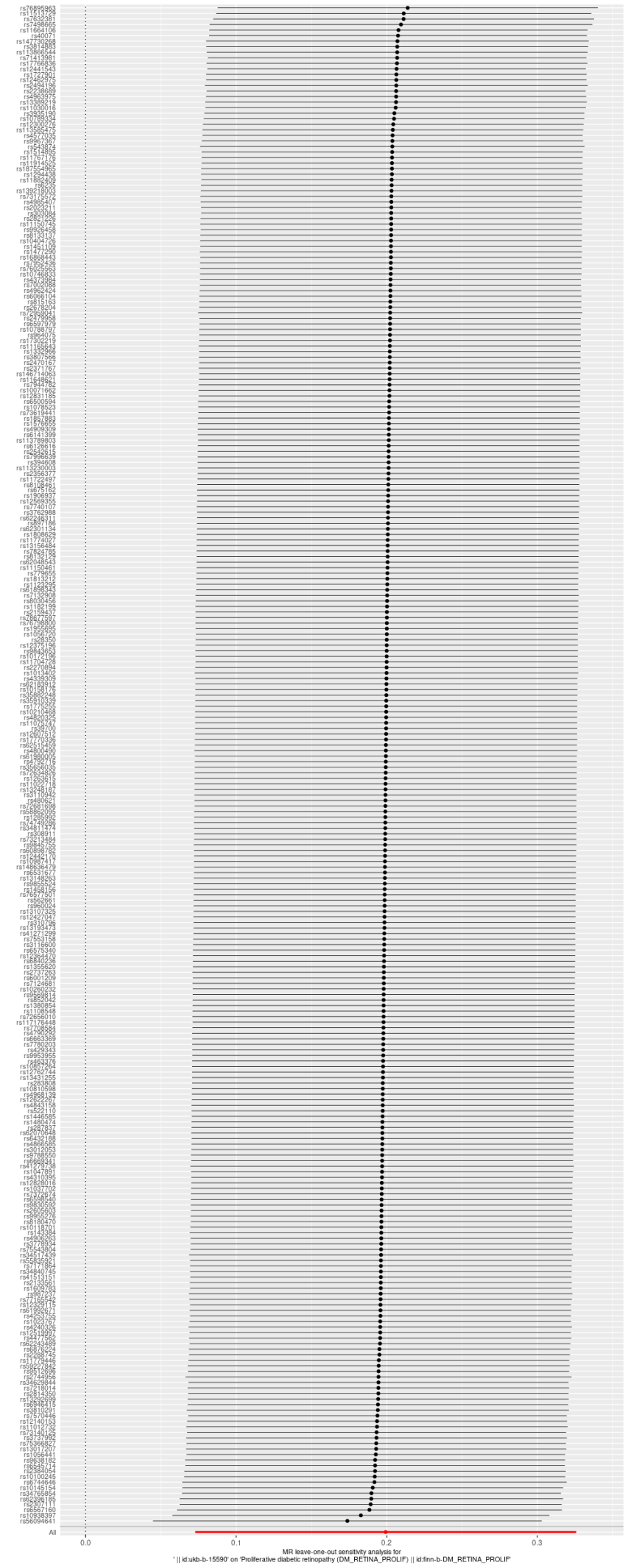

Supplement: Supplementary file 10 [file DataSheet_10.docx]
