# Supplementary material for: The causal effect of obesity on diabetic retinopathy: A two-sample Mendelian randomization study
Source: Front Endocrinol (Lausanne). 2023 Apr 3;14:1108731. doi: 10.3389/fendo.2023.1108731 (PMC10106681; doi:10.3389/fendo.2023.1108731)
Supplement: Supplementary file 11 [file Table_1.docx]

Table 1: The Information of Rucker'Q and Q-Q'.

| Exposures | Outcomes |  | Cochrane's Q | | | Rucker's Q' | | | Q-Q' |  |  |
| --- | --- | --- | --- | --- | --- | --- | --- | --- | --- | --- | --- |
|  |  | NSNVs | Q | df | P value | Q | df | P value | Q-Q' | df | P value |
| BMI | DR | 305 | 301.82 | 304 | 0.525 | 301.68 | 303 | 0.511 | 0.14 | 1 | 0.708 |
| Waist circumference |  | 252 | 292.95 | 251 | 0.036 | 292.45 | 250 | 0.034 | 0.5 | 1 | 0.479 |
| Hip circumference |  | 275 | 328.21 | 274 | 0.014 | 327.55 | 273 | 0.013 | 0.66 | 1 | 0.417 |
|  |  |  |  |  |  |  |  |  |  |  |  |
| BMI | Backgroud  DR | 306 | 334.57 | 305 | 0.118 | 333.86 | 304 | 0.115 | 0.71 | 1 | 0.399 |
| Waist circumference |  | 252 | 278.93 | 251 | 0.109 | 278.39 | 250 | 0.105 | 0.54 | 1 | 0.462 |
| Hip circumference |  | 275 | 328.89 | 274 | 0.013 | 328.72 | 243 | 0.012 | 0.17 | 1 | 0.68 |
|  |  |  |  |  |  |  |  |  |  |  |  |
| BMI | Proliferative  DR | 305 | 326.84 | 304 | 0.176 | 326.13 | 303 | 0.173 | 0.71 | 1 | 0.399 |
| Waist circumference |  | 252 | 285.42 | 251 | 0.067 | 285.41 | 250 | 0.061 | 0.01 | 1 | 0.92 |
| Hip circumference |  | 274 | 332.31 | 273 | 0.008 | 331.91 | 272 | 0.007 | 0.4 | 1 | 0.527 |

DR: diabetic retinopathy, BMI: body mass index, NSNVs: number of single-nucleotide variations.

Table 2： Mendelian randomization results of inverse variance weighted, weighted median and MR–Egger methods.

| Exposures | Outcomes | Inverse varianse weighted | | | | Weighted median | | | | MR-Egger | | | |
| --- | --- | --- | --- | --- | --- | --- | --- | --- | --- | --- | --- | --- | --- |
|  |  | OR | 95%LCI | 95%UCI | P Value | OR | 95%LCI | 95%UCI | P Value | OR | 95%LCI | 95%UCI | P Value |
| BMI | DR | 1.239 | 1.134 | 1.353 | 1.94E-06 | 1.268 | 1.102 | 1.458 | 8.87E-04 | 1.19 | 0.948 | 1.495 | 1.34E-01 |
| Waist circumference |  | 1.402 | 1.242 | 1.584 | 5.12E-08 | 1.397 | 1.158 | 1.686 | 4.89E-04 | 1.497 | 1.074 | 2.088 | 1.80E-02 |
| Hip circumference |  | 1.088 | 0.982 | 1.207 | 1.06E-01 | 1.167 | 1.008 | 1.351 | 3.90E-02 | 1.188 | 0.989 | 1.553 | 2.08E-01 |
| Hip circumference**^a^** |  | 1.107 | 1.003 | 1.221 | 4.20E-02 | 1.168 | 1.01 | 1.35 | 3.60E-02 | 1.21 | 0.938 | 1.562 | 1.43E-01 |
|  |  |  |  |  |  |  |  |  |  |  |  |  |  |
| BMI | Background  DR | 1.625 | 1.285 | 2.057 | 5.24E-05 | 1.793 | 1.247 | 2.579 | 1.63E-03 | 1.29 | 0.703 | 2.369 | 4.12E-01 |
| Waist circumference |  | 2.085 | 1.54 | 2.823 | 2.00E-06 | 2.297 | 1.439 | 3.667 | 4.88E-04 | 2.737 | 1.196 | 6.263 | 1.90E-02 |
| Hip circumference |  | 1.355 | 1.048 | 1.753 | 2.00E-02 | 1.541 | 1.034 | 2.218 | 3.30E-02 | 1.53 | 0.783 | 2.986 | 2.14E-01 |
| Hip circumference**^b^** |  | 1.394 | 1.085 | 1.791 | 9.00E-03 | 1.517 | 1.045 | 2.203 | 2.90E-02 | 1.567 | 0.817 | 3.001 | 1.78E-01 |
|  |  |  |  |  |  |  |  |  |  |  |  |  |  |
| BMI | Proliferative  DR | 1.401 | 1.247 | 1.575 | 1.46E-08 | 1.562 | 1.291 | 1.89 | 4.55E-06 | 1.249 | 0.924 | 1.688 | 1.49E-01 |
| Waist circumference |  | 1.696 | 1.455 | 1.977 | 1.47E-11 | 1.979 | 1.53 | 2.56 | 2.01E-07 | 1.734 | 1.14 | 2.638 | 1.00E-02 |
| Hip circumference |  | 1.182 | 1.033 | 1.351 | 1.50E-02 | 1.314 | 1.084 | 1.594 | 5.00E-03 | 1.291 | 0.911 | 1.831 | 1.52E-01 |
| Hip circumference**^c^** |  | 1.221 | 1.076 | 1.385 | 2.00E-03 | 1.318 | 1.093 | 1.591 | 4.00E-03 | 1.335 | 0.961 | 1.853 | 8.60E-02 |

DR: diabetic retinopathy, BMI: body mass index, ^a^one significant outlier (SNV: rs7903146) was deleted, ^b^one significant outlier (SNV:rs35506085) was deleted, ^c^two significant outliers (SNV:rs35506085; SNV: rs7903146) were deleted. 95%LCI: lower limit of 95% CI, 95%UCI: upper limit of 95% CI.
